# Analyzing the Role of Repolarization Gradients in Post-infarct Ventricular Tachycardia Dynamics Using Patient-Specific Computational Heart Models

**DOI:** 10.3389/fphys.2021.740389

**Published:** 2021-09-30

**Authors:** Eric Sung, Adityo Prakosa, Natalia A. Trayanova

**Affiliations:** ^1^Department of Biomedical Engineering, Johns Hopkins University, Baltimore, MD, United States; ^2^Alliance for Cardiovascular Diagnostic and Treatment Innovation, Johns Hopkins University, Baltimore, MD, United States

**Keywords:** ischemic cardiomyopathy, ventricular tachycardia, repolarization heterogeneity, computer modeling, personalized cardiac model

## Abstract

**Aims:** Disease-induced repolarization heterogeneity in infarcted myocardium contributes to VT arrhythmogenesis but how apicobasal and transmural (AB-TM) repolarization gradients additionally affect post-infarct VT dynamics is unknown. The goal of this study is to assess how AB-TM repolarization gradients impact post-infarct VT dynamics using patient-specific heart models.

**Method:** 3D late gadolinium-enhanced cardiac magnetic resonance images were acquired from seven post-infarct patients. Models representing the patient-specific scar and infarct border zone distributions were reconstructed without (baseline) and with repolarization gradients along both the AB-TM axes. AB only and TM only models were created to assess the effects of each ventricular gradient on VT dynamics. VTs were induced in all models *via* rapid pacing.

**Results:** Ten baseline VTs were induced. VT inducibility in AB-TM models was not significantly different from baseline (*p*>0.05). Reentry pathways in AB-TM models were different than baseline pathways due to alterations in the location of conduction block (*p*<0.05). VT exit sites in AB-TM models were different than baseline VT exit sites (p<0.05). VT inducibility of AB only and TM only models were not significantly different than that of baseline (*p*>0.05) or AB-TM models (*p*>0.05). Reentry pathways and VT exit sites in AB only and TM only models were different than in baseline (*p*<0.05). Lastly, repolarization gradients uncovered multiple VT morphologies with different reentrant pathways and exit sites within the same structural, conducting channels.

**Conclusion:** VT inducibility was not impacted by the addition of AB-TM repolarization gradients, but the VT reentrant pathway and exit sites were greatly affected due to modulation of conduction block. Thus, during ablation procedures, physiological and pharmacological factors that impact the ventricular repolarization gradient might need to be considered when targeting the VTs.

## Introduction

Post-infarct ventricular tachycardia (VT) is a life-threatening arrhythmia that increases the morbidity and mortality of patients with ischemic cardiomyopathy (ICM; Guandalini et al., 2019). Despite significant improvements in clinical therapies such as catheter ablation (a mainstay of VT treatment; Guandalini et al., 2019), VT recurrence remains unacceptably high (Tung et al., 2015; Marchlinski et al., 2016) in part due to an incomplete understanding about the complex pathophysiology of the arrhythmogenic substrate. Improved mechanistic understanding about the post-infarct VT substrate is critical to advancing VT treatment.

Reentrant VT arrhythmogenesis requires both conduction slowing and unidirectional conduction block. Repolarization dispersion, which affects unidirectional conduction block, has long been recognized as a contributing factor to VT arrhythmogenesis (Coronel et al., 2009; Opthof et al., 2009). Repolarization gradients, changes in repolarization along a specific axis, are also known to exist in the human heart (Coronel et al., 2009; Glukhov et al., 2010) and can also be exacerbated during sympathetic stimulation (Selvaraj et al., 2009; Vaseghi et al., 2012; Ajijola et al., 2017) either physiologically or pharmacologically. Furthermore, ventricular repolarization gradients have been observed to be steeper in ICM patients with VT than ICM patients without VT (Chauhan et al., 2006). Although it is well understood that repolarization heterogeneity from disease remodeling is present and important in VT arrhythmogenesis, it is unknown how or whether repolarization gradients influence post-infarct VT dynamics.

Biophysically detailed, computational modeling can provide mechanistic insights into VT arrhythmogenesis and dynamics that may not be easily discernible from experimental or clinical studies (Trayanova, 2011). Whole-heart models have been used to assess the mechanistic role of repolarization gradients on the generation of correct T-wave morphology (Keller et al., 2012). Furthermore, patient-specific whole-heart models that incorporate disease-remodeled repolarization heterogeneities have been used with great success in risk stratification of arrhythmic outcomes (Arevalo et al., 2016) and prediction of VT ablation targets (Prakosa et al., 2018; Sung et al., 2020).

The goal of the present study is to evaluate the impact of ventricular apicobasal and transmural (AB-TM) repolarization gradients on patient-specific, post-infarct VT dynamics (inducibility, VT exit site, and the reentry pathway) using computational whole-heart models. These findings would provide useful clinical insights for ablation therapy because these repolarization gradients are likely to be modulated by the patient’s sympathetic tone, often altered intraprocedurally by physiological or pharmacological means (Selvaraj et al., 2009; Vaseghi et al., 2012; Ajijola et al., 2017). The resulting repolarization gradients could impact post-infarct VT dynamics, which in turn would affect substrate characterization and where ablation lesions are delivered. Thus, understanding the interplay between ventricular AB-TM repolarization gradients and post-infarct VT could ultimately inform and improve ablation strategies.

## Materials and Methods

Data were appropriately de-identified and obtained in compliance with our institutional review board. Data presented here are available from the corresponding author upon reasonable request.

**Figure 1:**
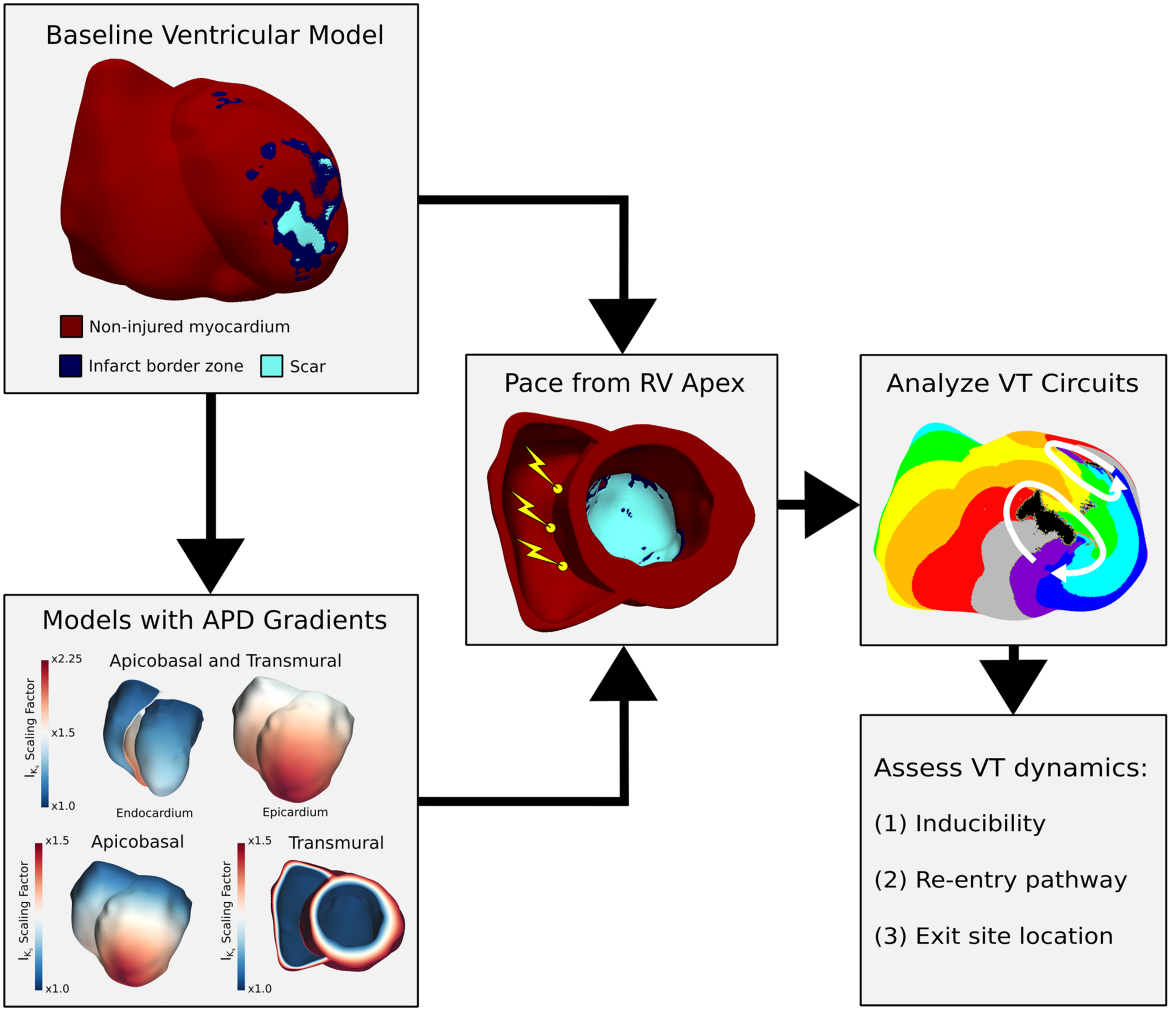
Study workflow. From imaging, baseline heart models were reconstructed with the patient-specific distribution of infarct border zone and scar. Models with repolarization gradients were constructed from baseline heart models by scaling I_Ks_ along the apicobasal and transmural (AB-TM) axes. Rapid pacing to induce VT was applied from three locations in the RV apex in all models. Activation maps were created for all induced VTs, and the resultant VT dynamics (inducibility, reentry pathway, and exit site location) were compared across different models. APD, action potential duration; I_Ks_, slowed delayed rectifier current; VT, ventricular tachycardia; RV, right ventricle.

The study workflow is presented in Figure 1. Briefly, baseline ventricular heart models with patient-specific scar and infarct border zone distributions were reconstructed from 3D late gadolinium-enhanced cardiac magnetic resonance imaging (LGE-CMR; Figure 1, top left panel). Models with ventricular repolarization gradients were created with and without action potential duration (APD) gradients along the apicobasal (AB) and transmural (TM) axes (Figure 1, bottom left panel). VTs were then induced in all models *via* rapid pacing (Figure 1, middle panel), and the resultant VT dynamics were compared in terms of inducibility, the reentrant pathway, and the exit site location (Figure 1, bottom right panel).

### Patient-Specific Computational Heart Model Construction

3D LGE-CMR images were acquired using a 1.5T clinical MRI scanner from 7 ICM patients without implanted cardioverter defibrillators (ICD). The resolution for six images was 0.625×0.625×2.5mm and 0.625×0.625×2.0mm for the last image. Each myocardium was then segmented with a semi-automated method as done in previous works using the variational implicit interpolation method (Prakosa et al., 2014, 2018; Arevalo et al., 2016; Sung et al., 2020). From the segmented myocardium, scar and infarct border zone were identified using the full width half max method as done in previous works (Schmidt et al., 2007; Arevalo et al., 2016; Prakosa et al., 2018). An example of a segmented short-axial LGE-CMR is shown in Supplementary Figure 1. Computational ventricular heart models were reconstructed from the segmented myocardium along with the patient-specific scar and infarct border zone distributions. To run simulations of cardiac electrical wave propagation, finite element, biventricular meshes were generated. More details about the computational model are presented in Supplementary Materials. Cardiac fiber orientations were assigned on an element-wise basis, as done in previous works (Arevalo et al., 2016; Prakosa et al., 2018; Sung et al., 2020), using a validated rule-based fiber generation method (Bayer et al., 2012) that is also consistent with human fiber orientations (Doste et al., 2019).

### Assigning Model Electrophysiological Properties

For baseline models, electrophysiological properties were assigned to non-injured myocardium and infarct border zone as done in previous works (Arevalo et al., 2016; Prakosa et al., 2018). Details on these electrophysiological properties are presented in Supplementary Materials. Scar was assumed to be non-conducting across all models. For models with repolarization gradients, APD gradients were created simultaneously along the apicobasal (AB) and transmural (TM) axes by scaling the slowly activated delayed rectifier potassium (I_Ks_) current in non-injured myocardium (Chiamvimonvat et al., 2017), as done in prior literature (Keller et al., 2012). The I_Ks_ current was modified because there is consistent evidence that supports a difference in I_Ks_ along both the transmural (Liu and Antzelevitch, 1995) and apicobasal (Szentadrassy et al., 2005) axes (Chiamvimonvat et al., 2017). Furthermore, there is clear evidence that beta-adrenergic stimulation effects on repolarization gradients are primarily mediated *via* changes to I_Ks_ (Volders et al., 2003; Harmati et al., 2011; Banyasz et al., 2014). These models with repolarization gradients along the AB and TM axes are referred to below as AB-TM models. The AB axis was determined by computing the geodesic distance of each mesh point from the base (Figure 1, bottom left panel). For the TM axis, the Laplace equation was solved with the endocardial and epicardial surfaces serving as the boundaries. The solution to the Laplace equation with these boundary conditions provided a smooth distance map from endocardium to epicardium regardless of the myocardial wall thickness (Figure 1, bottom left panel). From base to apex and from endocardium to epicardium, the I_Ks_ current was scaled by a factor of 1 to 1.5 in accordance with previous literature (Keller et al., 2012). Both scaling ranges were then multiplied together to achieve an I_Ks_ current scaling from 1 at the basal endocardium to 2.25 at the apical epicardium. This resulted in an APD at 90% repolarization (APD_90_) of 303ms at the basal endocardium, an APD_90_ at the basal epicardium and apical endocardium of 272ms, and lastly, an APD_90_ of 242ms at the apical epicardium. These APD_90_ values are consistent with those reported in clinical mapping studies for patients with ICM (Chauhan et al., 2006).

To evaluate the individual contributions the apicobasal (AB) and transmural (TM) repolarization gradients to changes in VT dynamics from baseline, separate ventricular models with only AB and only TM gradients were also reconstructed. For both AB and TM gradients, the I_Ks_ current was scaled by a factor from 1 to 1.5 along their respective axes in accordance with previous literature (Keller et al., 2012). Electrical wave propagation simulations were executed by solving the monodomain equations using the Cardiac Arrhythmia Research Package software.

### Baseline VT Induction Protocol

Baseline VTs were induced *via* pacing pulse trains, similar to standard clinical induction protocols used during ablation procedures (Koller et al., 1995). The pacing consisted of a series of six stimuli (S1) delivered at a fixed basic cycle length (BCL) followed by a premature stimulus (S2). This S2 stimulus was delivered at the earliest possible timing as dictated by the local tissue refractoriness at the pacing site (Koller et al., 1995). If after S2 no reentrant arrhythmia was observed, up to two subsequent premature stimuli, once again at the earliest possible timing, were delivered (S3, S4). The pacing location was chosen to be at the RV apex because this is where a pacing catheter would be placed during an ablation procedure (Koller et al., 1995). Furthermore, by only pacing from the RV apex, the effects of activation wavefront directionality which can have an impact on the arrhythmogenic substrate (Anter et al., 2020) were controlled for. To account for potential variability in where the pacing catheter could be placed, three locations were identified in the RV apex (Figure 1, middle panel). For each pacing location, two different S1 BCLs were tested (BCL=600ms and BCL=350ms) to assess the substrate arrhythmogenicity more comprehensively from limited pacing locations.

### VT Induction Protocol in Models With Repolarization Gradients

Stimulus protocols that successfully induced VT in baseline ventricular models were then applied to AB-TM models (Anter et al., 2020) Because the APD changes alter the tissue effective refractory period, the earliest possible timings of S2-S4 are likely to change for models with APD gradients (Koller et al., 1995). Thus, to account for this change, two induction protocols were considered for AB-TM models. First, the exact timings of S2-S4 from protocols that induced VTs in baseline models were re-applied to AB-TM models. This first protocol was referred to as the exact timed stimulus (XTS) protocol. Second, the earliest possible timings of S2-S4 were identified and applied in AB-TM models. This second protocol was referred to as the earliest timed stimulus (ETS) protocol. Both ETS and XTS protocol timings of S1, S2, S3, and S4 for baseline and all APD gradient models are shown in Supplementary Table 1. These same stimulus protocols were applied to AB only and TM only models as well to dissect the contributions of each individual repolarization gradient on VT dynamics.

### Analysis of VT Circuits

Activation maps were created for all induced VTs across all models. VTs were defined as having at least two reentrant cycles, the same definition as used in previous works (Arevalo et al., 2016; Prakosa et al., 2018; Sung et al., 2020). To better visualize the VT circuit, activation times were divided into 8 separate isochrones with gray being the earliest activation and purple representing the latest activation (Figure 1, right panel). The latest activation (purple) was defined to be at the timing at which the wavefront exited the circuit using a similar definition and color scheme as done in prior literature (Tung et al., 2020). Reentry pathways were manually traced by inspecting the activation maps and starting from and ending at the latest activation (purple). VT exit sites were manually annotated as the location at which the wavefront exited the circuit.

VT dynamics (inducibility, reentry pathway, and exit site location) were compared between models. Reentry pathways across models were considered different if there was a difference in the conduction pathway. VT exit sites were considered similar between models if the locations of the VT exit site were spatially concordant between circuits.

### Statistical Analysis

Because the sample size was small, Fisher’s exact test was used to assess for whether VT dynamics (inducibility, reentry pathway, and exit site location) matched or did not match across all models. Comparisons were made between VT dynamics in baseline and AB-TM models, baseline and AB only models, baseline and TM only models, AB-TM and AB only models, and AB-TM and TM only models.

## Results

### Characteristics of VT Dynamics in Baseline Models

Table 1 provides a summary of the baseline ventricular models and the number of VTs induced across models. In all models, scar and infarct border zone distributions localized to the anterior and apical regions of the left ventricle. The mean scar volume was 12.6±5.0% and the mean infarct border zone volume was 6.8±1.9% (Table 1).

**Table 1:** Summary of the baseline ventricular models and the number of VTs induced across models.

Model	Infarct Distribution	Scar (%)	Infarct Border Zone (%)	Number of VTs	Corresponding VTs
A	Anterior	11.7	6.7	1	VT 1
B	Anterior	12.7	7.6	1	VT 2
C	Anterior	18.8	9.7	3	VT 3, VT 4, VT 5
D	Anterior	7.0	3.5	2	VT 6, VT 7
E	Anterior	19.4	7.0	1	VT 8
F	Apex	11.5	5.6	1	VT 9
G	Apex	7.0	7.5	1	VT 10

*Infarct distribution refers to the primary locations of scar and infarct border zone distributions. Corresponding VTs refers to the baseline VTs induced in the model. VT: ventricular tachycardia*.

**Figure 2:**
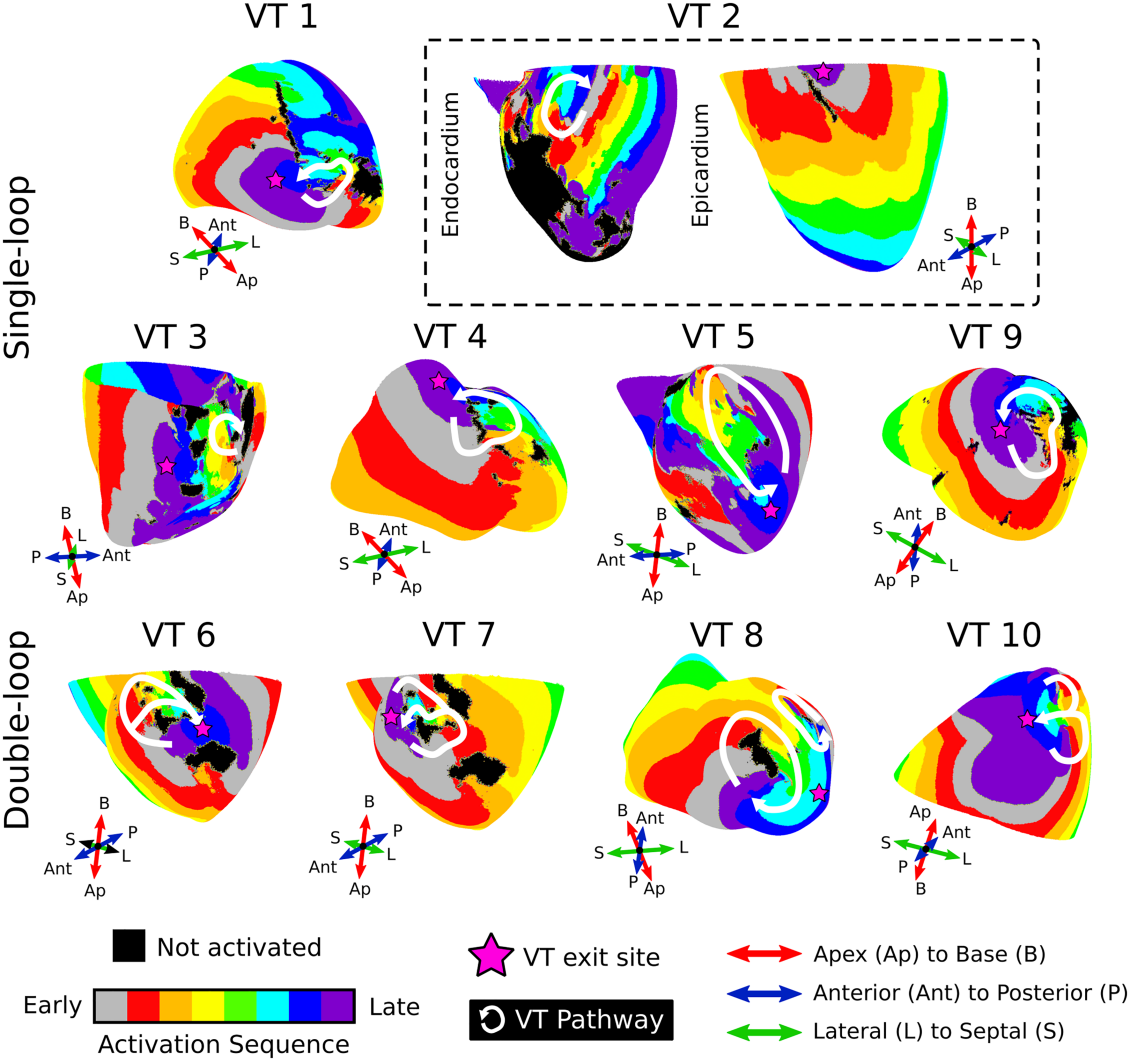
VT morphologies in baseline models. Displayed are activation maps of the 10 induced VT circuits. Activation maps were created such that the latest activation (purple) represents the time at which the wavefront has exited the circuit. The reentry pathway is denoted in white, and the pink start denotes the estimated location of the VT exit site. The first two rows demonstrated examples of single-loop morphologies; the last row shows the double-loop morphologies in baseline models. For VT 2 (dashed box), two views are shown to fully visualize the VT circuit. VT, ventricular tachycardia; Ap, apex; B, base; Ant, anterior; P, posterior; L, lateral; S, septal.

Ten VTs were induced across the seven baseline models (Table 1). The median (interquartile range) number of VTs per model was 1 (0.75). The baseline VT morphologies are displayed in Figure 2. The VT circuit locations and sizes were highly variable across models and dependent on the patient-specific substrate geometry. There were two predominant morphology patterns observed: baseline VTs 1, 2, 3, 4, 5, and 9 all exhibited a single-loop-type morphology whereas baseline VTs 6, 7, 8 and 10 all exhibited a double-loop- or figure-of-eight-type morphology. Lastly, VT exit sites were not always located directly along the reentrant pathway (e.g., VT 2 and VT 3).

### VT Inducibility Was Similar Between AB-TM and Baseline Models

In AB-TM models, seven VTs were induced across both the ETS and XTS protocols. The inducibility of AB-TM models was not significantly different than the inducibility of baseline models (*p*>0.05). Moreover, all induced VT circuits in AB-TM models localized to the same region of the heart and involved a similar common pathway as their respective baseline VT circuits. These results suggest that repolarization dispersion may not have a pronounced impact on arrhythmogenic propensity of the substrate nor the location of the VT; rather, these factors may be directly related to the patient-specific substrate distribution.

### Repolarization Gradients Affect Reentrant Pathways by Altering the Locations of Conduction Block

For both XTS and ETS protocols, VTs induced in AB-TM models encompassed different reentrant pathways than VTs induced in baseline models (*p*<0.05). For the AB-TM models, 6/7 of the VTs had different conduction pathways.

**Figure 3:**
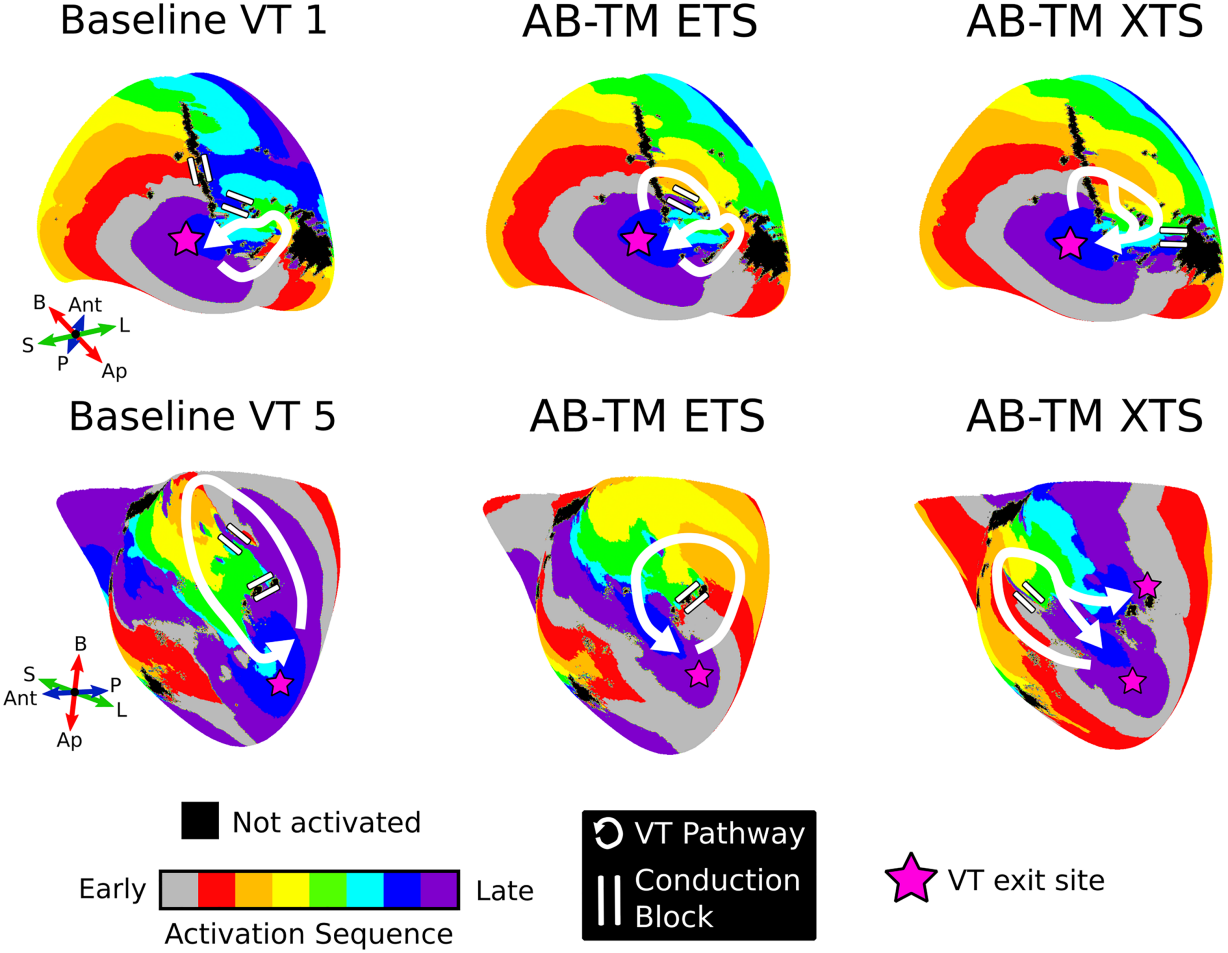
Examples where APD gradients altered the VT pathway without affecting the VT exit site location. For baseline VTs 1 and 5, APD gradients did not significantly alter the VT exit site but did change the reentry pathway by modulating the location of conduction block. The VT pathway is shown in white, areas of conduction block are shown with two white lines, and the VT exit sites are denoted with pink stars. ETS, earliest timed stimulus; XTS, exact timed stimulus; VT, ventricular tachycardia; APD, action potential duration; AB, apicobasal; TM, transmural; Ap, apex; B, base; Ant, anterior; P, posterior; L, lateral; S, septal.

Figure 3 presents two examples of how repolarization gradients affect the VT reentrant pathway by changing locations of conduction block. For instance, baseline model VT 1 was a single-loop morphology with two areas of conduction block (Figure 3, top left). In AB-TM models, both the ETS and XTS protocols induced VTs with double-loop-type morphologies that had the same exit site as the baseline VT but utilized different pathways that were blocked in the baseline model (Figure 3, top center and right).

Baseline model VT 5 was a single-loop morphology with a large macro-reentrant pathway (Figure 3, bottom left). The AB-TM ETS-induced VT circuit was smaller and used a pathway that was blocked in the baseline model (Figure 3, bottom center). The AB-TM XTS-induced VT localized to the same region as the baseline model VT 5, but used a different pathway and additionally had a second exit site (Figure 3, bottom right). These examples demonstrate how repolarization gradients in conjunction with premature stimulus timing modulate unidirectional conduction block, and hence determine the resultant reentrant pathway.

### VTs in Models With Repolarization Gradients Had Different Exit Sites Than the Corresponding Baseline Model VTs

**Figure 4:**
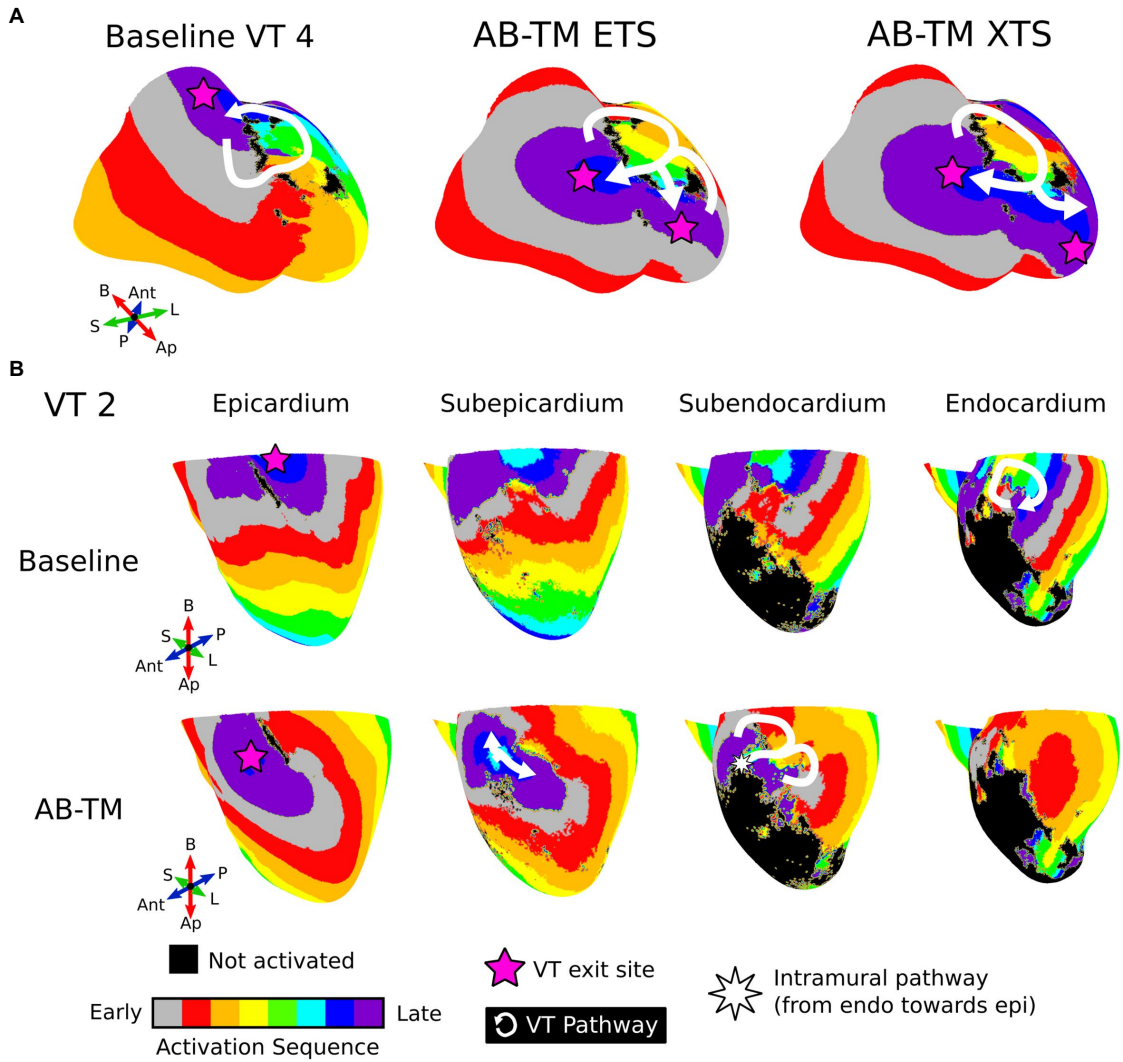
Examples where APD gradients altered both the VT reentrant pathway and exit site. **(A)** Illustration of APD gradient reversing VT 4’s entrance and exit site. White denotes the reentry pathway, and the pink star denotes the VT exit site. **(B)** Illustration of intramural VT 2 circuit. Each column denotes different depths in the myocardium. The VT pathway is drawn with the white arrow and the pink star denotes the exit site. The white star denotes a point at which the pathway travels intramurally in the direction from the endocardium to the epicardium. ETS, Earliest timed stimulus; XTS, Exact timed stimulus; VT, ventricular tachycardia; APD, action potential duration; AB, apicobasal; TM, transmural; Ap, apex; B, base; Ant, anterior; P, posterior; L, lateral; S, septal.

Most VT exit sites in AB-TM models were different than VT exit sites in baseline models. Whereas 3/6 AB-TM ETS-induced VTs had similar VT exit sites as baseline VTs, only 2/7 AB-TM XTS-induced VTs had the same exit site as baseline VTs. Figure 4 shows examples of how repolarization gradients altered VT exit sites. Baseline VT 4 had a single-loop morphology that entered through the mid interventricular junction and exited toward the right ventricular outflow tract (Figure 4A, left). However, VT 4’s entrance and exit sites were reversed in the AB-TM model (Figure 4A, center and right).

Baseline VT 2 was a single-loop morphology with a fully endocardial circuit and exit site at the basal epicardium (Figure 4B, first row). In the AB-TM model, the ETS protocol did not induce VT; the XTS protocol induced a novel figure-of-eight VT morphology with a purely intramural circuit (Figure 4B, second row). Although this VT utilized similar conducting channels, it had a different exit site displaced more inferiorly (Figure 4B, bottom row). These results highlight how repolarization gradients can predispose the VT circuit to use specific exit sites, due to the location of conduction block.

### AB Only or TM Only Models Have Similar VT Inducibility as AB-TM and Baseline Models

To better understand the contributions of the individual AB and TM APD gradients to VT dynamics, we applied the same induction protocols to AB only and TM only APD gradient models. Supplementary Figure 2 provides a comprehensive illustration of the VTs induced across all baseline, AB-TM, AB, and TM models.

VT inducibility of AB and TM models was not significantly different than inducibility of AB-TM models (*p*>0.05) nor baseline models (*p*>0.05). Among AB models across both ETS and XTS protocols, eight VTs were induced. For both ETS and XTS protocols in TM models, eight VTs were also induced. Concordantly, all induced VTs in AB and TM models also utilized similar common pathways as VTs in both baseline and AB-TM models. These results further emphasize how repolarization gradients do not significantly affect VT inducibility nor the location of induced VT circuits.

### The Presence of Either AB or TM Repolarization Gradients Alone Is Sufficient to Alter Reentrant Pathways and VT Exit Sites

**Figure 5:**
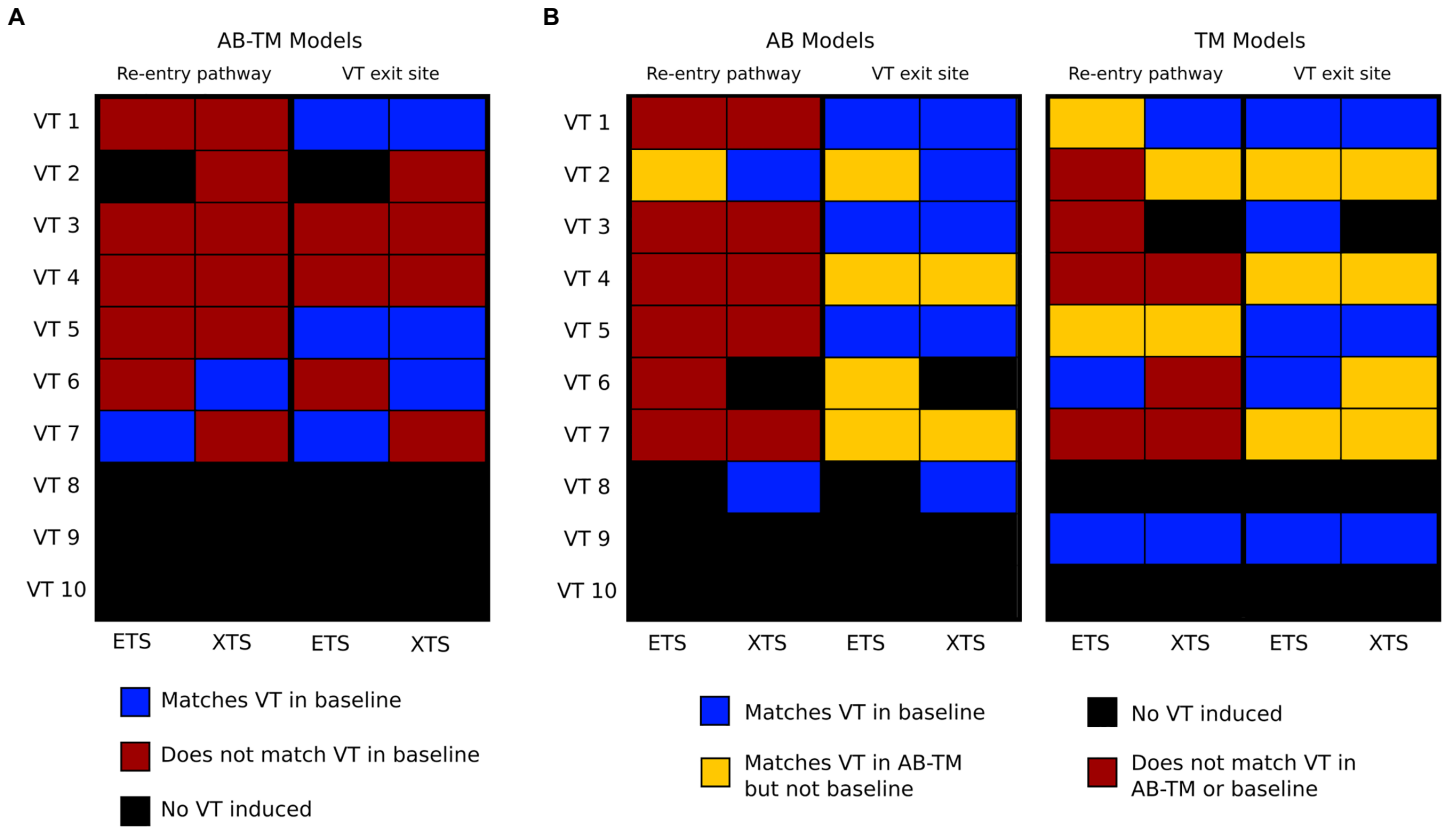
Summary of reentry pathway and VT exit sites across all models with repolarization gradients. **(A)** Comparisons of reentry pathway and VT exit sites between AB-TM and baseline models. Each row represents a different VT. Blue means the two were consistent, red means that the two were not consistent, and black means no VT was induced. **(B)** Comparisons of reentry pathway and VT exit sites between AB and TM with baseline and AB-TM models. Blue means there was a match with baseline models, red means there was no match with either baseline or AB-TM models, orange means there was a match with AB-TM models but not baseline models, and black means there was no VT induced. VT, ventricular tachycardia; AB, apicobasal; TM, transmural.

An additional set of simulations were conducted to determine the separate roles and contributions of the AB and TM gradients in modifying VT circuits in the patients’ hearts. Both AB only and TM only gradients affected locations of conduction block which subsequently altered the reentrant pathway and VT exit site location. Figure 5A summarizes how VT dynamics in AB-TM models compare with VT dynamics in baseline models; Figure 5B summarizes how VT reentrant pathways in AB only and TM only models compare with reentrant pathways in AB-TM and baseline models. Reentrant pathways in both AB models and TM models were different than reentrant pathways in baseline models (*p*<0.05) and in AB-TM models (*p*<0.05).

Most VT exit sites in AB and TM models were at different locations than baseline VT exit sites but were spatially concordant with VT exit sites in AB-TM models (Figure 5B). AB ETS induced 4/7 VTs with different exit sites; contrarily, AB XTS induced only 2/7 VTs with different exit sites than baseline. For TM models, 3/8 ETS-induced VTs and 4/7 XTS-induced VTs had different exit sites than baseline. Thus, these results demonstrate how both independent AB and TM repolarization gradients are sufficient to alter post-infarct VT dynamics by modulating locations of conduction block.

### Models With Repolarization Gradients Revealed Multiple VT Morphologies That Manifested Within the Same Conducting Channels

**Figure 6:**
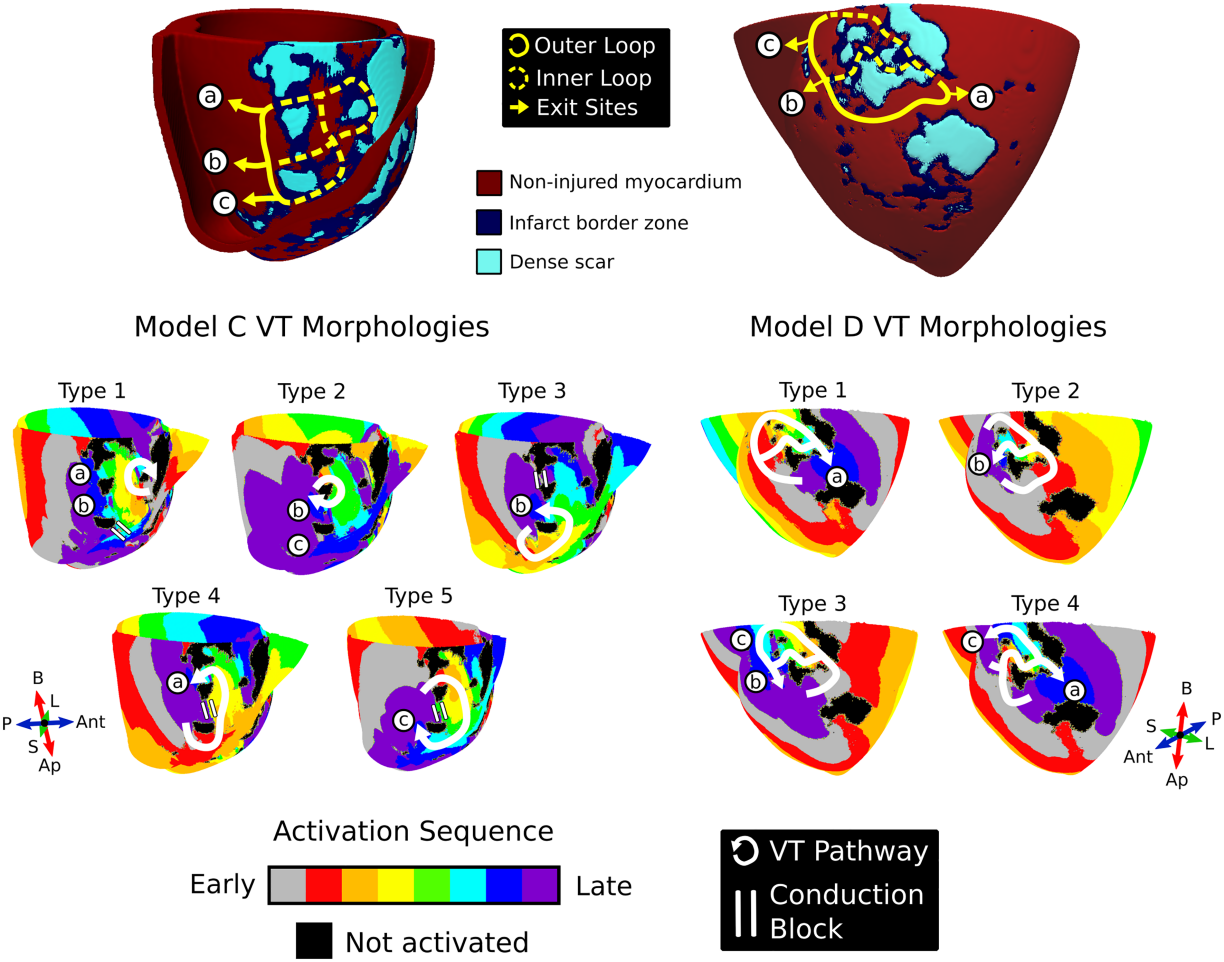
Repolarization gradients reveal multiple morphologies within the same conducting channels. *Top:* Models C and D were two examples where multiple VT morphologies manifested in the same conducting channels. The yellow lines denote the possible conduction pathways in the substrate; the solid yellow line is the outer loop, the dashed yellow line is the inner loop, and the arrowhead denotes the exit site. For both models, all exit site locations are denoted with the circled letters “a,” “b,” and “c.” *Bottom:* The distinct VT morphologies induced in the substrate across models C and D. White arrows denote the reentrant pathway, and the double white lines refers to regions of conduction block. VT, ventricular tachycardia; Ap, apex; B, base; Ant, anterior; P, posterior; L, lateral; S, septal.

Figure 6 illustrates two models where multiple VT morphologies were uncovered within the same conduction channels through the various APD gradients and stimulus protocols. In model C, five different morphology types were identified (Figure 6, left). The first type of VT morphology had a reentrant pathway completely contained within the inner loop of the circuit and exited through both sites “a” and “c” (Figure 6, left). The morphology type 2 pathway involved a small single loop that rotated about a patch of dense scar proximal to exit site “b” in the mid septum also through site “c” (Figure 6, left). Here, the third morphology VT pathway entered through site “c,” was blocked at site “a,” and exited through site “b” (Figure 6, left). VT type 4 entered through site “c,” was blocked at site “b,” and exited through site “a” whereas type 5 had the reversed chirality, blocking at site “b,” but entering through site “a” and exiting through site “c” (Figure 6, left).

For model D, morphology types 1 and 2 were the most common VTs observed (Figure 6, right). The first VT type entered through both sites “b” and “c” and exited *via* site “a.” On the other hand, the second morphology entered through sites “a” and “c” and exited through site “b” in a figure-of-eight configuration. The final two morphologies had the reversed chirality of the first two morphology types. The third VT morphology represented the reversed chirality of morphology type 1 with the entrance at site “a” and exits at both sites “b” and “c.” Lastly, the fourth VT morphology represented the reversed chirality of morphology type 2, entering only through site “b” and exiting through both sites “a” and “c.”

Both models C and D demonstrate how the correct combination of repolarization gradients and premature stimulus timing can result in multiple VT morphologies manifesting within the same conducting channels in the patient-specific substrate.

## Discussion

This study assessed the effects of AB-TM repolarization gradients, in the form of APD gradients, on post-infarct VT dynamics using computational whole-heart models. Inclusion of repolarization gradients did not significantly affect VT inducibility. However, in most patient-specific substrates, these repolarization gradients altered the VT reentrant pathway and the VT exit site location by modulating locations of unidirectional conduction block. Lastly, incorporating repolarization gradients unmasked multiple VT morphologies within the same conducting channels, highlighting the complex interdependence between electrophysiological heterogeneity and the patient-specific scar and infarct border zone distribution.

Catheter ablation, one of the major therapies for VT, relies on careful understanding of the post-infarct VT circuit to successfully eliminate substrate arrhythmogenicity (Guandalini et al., 2019). Modulation of sympathetic tone such as with anesthesia, sympathomimetics, and stress can all impact ventricular electrophysiology and arrhythmogenicity (Selvaraj et al., 2009; Vaseghi et al., 2012; Ajijola et al., 2017). Increased sympathetic stimulation modulates the repolarization gradient *via* decreases in APD (Selvaraj et al., 2009; Vaseghi et al., 2012; Ajijola et al., 2017), and increases spatial dispersion of repolarization in infarcted hearts where sympathetic denervation is heterogeneous (Ajijola et al., 2017). Unsurprisingly, ICM patients with VT exhibit larger repolarization gradients than ICM patients without VT (Chauhan et al., 2006). Our results offer mechanistic insights into how repolarization gradients can have a major impact on VT dynamics. Although the disease-remodeled, patient-specific substrate dictates arrhythmogenic propensity, repolarization gradients combined with premature stimulus timing modulate locations of unidirectional conduction block which in turn determines the reentrant pathway and the VT exit site, both of which are important considerations for ablation. Ablation strategies that target only a single part of the clinical VT circuit may neglect alternate pathways capable of harboring other distinct VTs that manifest under different electrical conditions. Thus, the patient’s current sympathetic state and repolarization dynamics, which could be assessed *via* analysis of the T-wave morphology on electrocardiogram (ECG; Draisma et al., 2006; Ramirez et al., 2011) or estimation of local repolarization properties *via* intraprocedural mapping (Yue et al., 2004), should be considered during VT ablation to ensure that all possible, relevant VTs are eliminated.

Our results also further echo how VT circuits are not constant entities and how analysis of structural heterogeneities alone is unlikely to be sufficient in characterizing the post-infarct substrate for ablation therapy. Concomitant sinus rhythm and VT activation mapping studies provide clear evidence that lines of conduction block are not always fixed (Rottmann et al., 2019). Prior studies implicate the propensity for multiple VT morphologies to manifest in the same location and utilize similar conduction pathways (Anter et al., 2016, 2018). Collectively, these studies emphasize how VT circuits result from a combination of both fixed and functional heterogeneities. Concordantly, we observed how multiple VT morphologies anchored to the same location not only because of the scar and infarct border zone geometry, but also because of the locations of conduction block as dictated by the repolarization gradients and premature stimulus timing. Our study highlights how the synergistic interplay between functional (repolarization gradients, stimulus timing) and structural heterogeneities (patient-specific scar and infarct border zone distribution) needs to be considered during ablation to successfully target all VTs.

Our study also has implications for noninvasive, computationally driven approaches in the clinic. Incorporation of APD gradients is unlikely to significantly affect virtual electrophysiological studies for arrhythmia risk stratification (Arevalo et al., 2016) because VT inducibility in the models was overall not significantly affected by the repolarization gradients. However, repolarization gradients may impact the location of virtual-heart ablation lesions. Prior strategies for targeting involved delivering minimal-sized lesions at locations with the greatest arrhythmogenic propensity (Prakosa et al., 2018; Sung et al., 2020). Our study demonstrates how repolarization gradients can give rise to distinct VT morphologies which would change the proposed ablation targets. However, unlike the present study, previous studies evaluated arrhythmogenic propensity using multiple pacing locations from across the left ventricle. One such study that relied on collective analysis of results from multiple pacing site locations determined that virtual-heart VT ablation predictions were robust to changes in several electrophysiological parameters (Deng et al., 2019). The directionality of wavefront activation affects the arrhythmogenic substrate (Anter et al., 2020) and could give rise to distinct VT morphologies. Hence the VT morphologies that were uncovered in models with repolarization gradients could potentially have been uncovered in baseline models using different pacing locations not at the RV apex.

Our study also suggests that accurate prediction of VT exit sites with computational heart models is likely to be affected. In a recent study, virtual-heart VT circuits were found to be spatially concordant with ECG-based localization of VT exit sites (Zhou et al., 2021). In one instance, the virtual-heart VT entrance site matched the clinical ECG-based VT exit site prediction, hinting that the predicted virtual-heart VT circuit was at the same location as the clinical VT, but with an opposite axis. The current study indicates that this discrepancy could be explained by the presence of a repolarization gradient because this alone was shown to be sufficient to reverse VT entrance and exit sites. Thus, further work investigating virtual heart-based VT exit site localization should consider the role of repolarization gradients and other electrical heterogeneities.

## Limitations

Our study has several limitations. First, the arrhythmic outcome was unknown in these seven patients, and it is unknown whether these patients underwent a VT ablation procedure. The 3D LGE-CMR scans were obtained prior to ICD implantation and there was no subsequent follow-up. Secondly, we did not have access to the patient ECG data, and thus could not use this information to create personalized, AB-TM ventricular repolarization gradients or to localize the VT morphology as a form of validation. This lack of clinical electrophysiological data also precluded a comprehensive personalization of electrophysiological properties for the non-injured myocardium and infarcted tissues in the whole-heart models. Future studies should include patient cohorts with electroanatomical mapping data and/or ECG data for clinical validation and comparison. Third, similar to other personalized ventricular modeling studies (Arevalo et al., 2016; Prakosa et al., 2018; Sung et al., 2020), we could not include the Purkinje system (which could also impact repolarization dispersion) because it is not possible to acquire its geometry from the LGE-CMR images alone. Fourth, as our goal was to assess the effects of AB-TM repolarization gradients on post-infarct VT dynamics, sympathetic innervation changes were not represented. Given our findings, it is possible that the resultant repolarization dispersion from this heterogeneous sympathetic denervation could have an additional impact on post-infarct VT dynamics. Another limitation is that we considered only AB-TM repolarization gradients because these corresponded with well-documented physiological gradients known to exist in the human heart. Other gradients are thought to exist which may result in a different effect on post-infarct VT dynamics. Lastly, we modified only I_Ks_ to create the AB-TM repolarization gradients. Other potassium currents are also present, and some may contribute to the repolarization gradients (Szentadrassy et al., 2005; Chiamvimonvat et al., 2017). However, our findings indicate that VT dynamics were modulated by conduction block which stems from repolarization heterogeneity. Hence, the conclusions of our study should not change significantly even if different potassium currents were modified so long as the changes to repolarization remain consistent.

## Conclusion

Using computational whole-heart models, we characterized the effects of various repolarization gradients on patient-specific, post-infarct VT dynamics. Although repolarization gradients did not impact VT inducibility, repolarization gradients altered both the VT pathway and VT exit site locations due to changes in the location of conduction block. This implies that repolarization gradients could impact post-infarct VT dynamics and would affect the ablation strategy. Thus, physiological and pharmacological factors that impact the repolarization gradient might need to be considered during ablation to optimize targeting of VTs.

## Data Availability Statement

The raw data supporting the conclusion of this article will be made available by the authors, without undue reservation.

## Ethics Statement

The studies involving human participants were reviewed and approved by Johns Hopkins School of Medicine Institutional Review Board. The patients/participants provided their written informed consent to participate in this study.

## Author Contributions

ES designed the study, and ran the simulations. AP reconstructed the models. ES wrote the original draft of the manuscript and prepared all tables and Figures. ES, AP, and NT all contributed to the manuscript revisions, read, and approved this submitted version.

## Funding

This work was supported by the National Institutes of Health (R01HL142496 and R01HL142893 to NT); and an American Heart Association Predoctoral Fellowship to ES.

## Conflict of Interest

The authors declare that the research was conducted in the absence of any commercial or financial relationships that could be construed as a potential conflict of interest.

## Publisher’s Note

All claims expressed in this article are solely those of the authors and do not necessarily represent those of their affiliated organizations, or those of the publisher, the editors and the reviewers. Any product that may be evaluated in this article, or claim that may be made by its manufacturer, is not guaranteed or endorsed by the publisher.
