# Supplementary material for: Analyzing the Role of Repolarization Gradients in Post-infarct Ventricular Tachycardia Dynamics Using Patient-Specific Computational Heart Models
Source: Front Physiol. 2021 Sep 30;12:740389. doi: 10.3389/fphys.2021.740389 (PMC8514757; doi:10.3389/fphys.2021.740389)
Supplement: Supplementary file 1 [file Data_Sheet_1.DOCX]

**Supplemental Materials**

**Title**: Analyzing the Role of Repolarization Gradients in Post-infarct Ventricular Tachycardia Dynamics Using Patient-Specific Computational Heart Models

**Authors**: Eric Sung BA^1,2^, Adityo Prakosa PhD^1,2^, Natalia A Trayanova PhD^1,2^

1 Department of Biomedical Engineering, Johns Hopkins University, Baltimore, MD, USA

2 Alliance for Cardiovascular Diagnostic and Treatment Innovation, Johns Hopkins University, Baltimore, MD, USA

**Supplemental Methods**

*Details on Computational Modeling*

Finite element biventricular meshes were created for all patients. Nodes in all meshes were then tagged as one of 3 regions: non-injured myocardium, infarct border zone, and dense scar. At each mesh node tagged as non-injured, the human ventricular myocyte ten Tusscher model was used to model the ionic currents.^1^ For the non-injured tissue regions, the conductivities were 0.08 and 0.009 S/m in the longitudinal and transverse directions, respectively. Mesh nodes tagged as dense scar were assumed to be non-conducting.

For nodes tagged as infarct border zone, the ionic currents were adjusted such that the peak I­_Na_ was scaled to 38%, I_CaL_ was scaled to 31%, I­_Kr_ was scaled to 30%, and I_Ks_ was scaled to 20%, as previously described.^2,3^ These changes resulted in a decrease in maximum upstroke velocity (non-injured 6.7 vs. border zone 11.6 V/sec) and a decrease in maximum voltage amplitude (non-injured 20 vs. border zone 35 mV) as was previously described.^3^ To represent remodeling changes that happen in infarct border zone, the transverse conductivity was decreased by 90%, as we have done in several of our previous works.^2–6^

To simulate electrical wave propagation, a system of partial differential equations called the monodomain equations^7^ were solved across the mesh domain in response to electrical stimuli that were applied to excite ~ 1 mm^3^ of ventricular tissue (current strength: 100 µA/cm^2^). All simulations were conducted using the Cardiac Arrhythmia Research Package (CARP) software.

**Supplemental Results**

AB-TM APD gradients eliminated the inducibility of VTs 8, 9, and 10. For VT 8, only the AB XTS protocol induced an identical VT morphology whereas all other APD gradients resulted in no VTs induced. The VT circuit was located in a region with greater wall thickness and thus was more affected by the TM APD gradient. For VT 9, only TM ETS and XTS protocols induced VTs. Here, the circuit for VT 9 localized to a region with wall thinning; hence the TM APD gradient likely did not significantly affect the electrophysiological heterogeneity in that region. On the other hand, AB gradients resulted in bidirectional conduction block in this circuit, preventing any sustained re-entrant activity. Lastly for VT 10, when any APD gradient was incorporated, the baseline VT morphology was not observed and was replaced by multiple non-sustained re-entrant cycles. The substrate distribution for model G was complex and thus, these electrophysiological changes de-stabilized any re-entrant arrhythmia that formed.

All VTs induced in APD gradient models used the same conducting channels as baseline VTs. However, in the case of except for the 3 baseline VTs 3, 4, and 5 that all belonged to model C, the protocols that induced the baseline VTs ended up inducing other VTs. For model C, the protocol that induced baseline VT 3, instead induced a VT morphology at the same location as baseline VT 5. The protocol that induced baseline VT 4, then induced a morphology that matched the location of baseline VT 3, and lastly, the protocol that induced baseline VT 5, induced a morphology that matched the location of baseline VT 4. Thus, although the XTS protocol induced different VTs in the AB-TM than the baseline model, the 3 induced morphologies still matched the location of other baseline VTs in the same model.

**Supplemental Figures/Tables**

| VT | Baseline/XTS | | | | AB ETS | | | | TM ETS | | | | AB-TM ETS | | | | |
| --- | --- | --- | --- | --- | --- | --- | --- | --- | --- | --- | --- | --- | --- | --- | --- | --- | --- |
|  | S1 (ms) | S2 (ms) | S3 (ms) | S4 (ms) | S1 (ms) | S2 (ms) | S3 (ms) | S4 (ms) | S1 (ms) | S2 (ms) | S3 (ms) | S4 (ms) | S1 (ms) | S2 (ms) | S3 (ms) | S4 (ms) |  |
| 1 | 600 | 330 |  |  | 600 | 310 |  |  | 600 | 300 |  |  | 600 | 290 |  |  |  |
| 2 | 350 | 280 | 330 |  | 350 | 270 |  |  | 350 | 270 | 330 | 280 | 350 | 260 |  |  |  |
| 3 | 600 | 330 | 330 | 310 | 600 | 320 | 330 | 300 | 600 | 310 | 320 |  | 600 | 300 |  |  |  |
| 4 | 600 | 330 | 310 |  | 600 | 320 | 340 |  | 600 | 350 |  |  | 600 | 300 |  |  |  |
| 5 | 350 | 280 |  |  | 350 | 290 |  |  | 350 | 330 |  |  | 350 | 260 |  |  |  |
| 6 | 600 | 330 | 330 | 270 | 600 | 320 | 250 | 300 | 600 | 310 | 250 |  | 600 | 300 | 250 | 270 |  |
| 7 | 600 | 330 | 300 | 290 | 600 | 310 | 280 |  | 600 | 300 | 310 |  | 600 | 290 | 320 | 260 |  |
| 8 | 600 | 330 | 240 | 260 | 600 | 310 | 200 | 450 | 600 | 300 | 200 | 340 | 600 | 300 | 220 | 220 |  |
| 9 | 350 | 250 | 410 |  | 350 | 220 | 360 | 350 | 350 | 240 |  |  | 350 | 240 | 370 | 290 |  |
| 10 | 350 | 250 | 550 | 270 | 350 | 250 | 500 |  | 350 | 230 | 370 | 440 | 350 | 150 | 400 |  |  |

**Supplemental Table 1: Stimulus protocols used to induce VTs in baseline and APD gradient models.** The first column (baseline) lists the protocols used to induce baseline VTs. These same protocols, which we call the exact timed stimulus (XTS) protocols, were also used to induce VTs in AB, TM, and AB-TM models. Columns 2 through 4 list the earliest timed stimuli (ETS) protocols used to induce VTs in AB, TM, and AB-TM models. AB: Apicobasal, TM: transmural

**
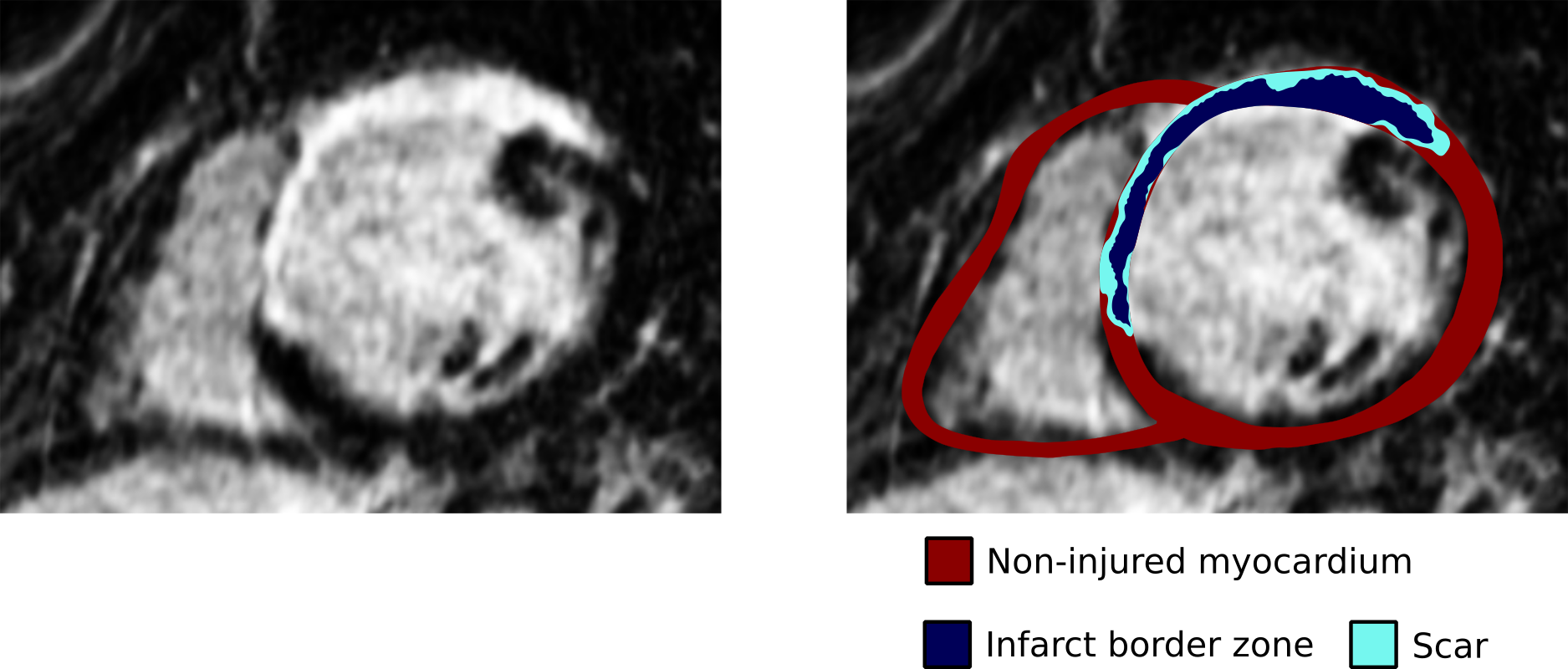
**

**Supplemental Figure 1: Example of a segmented 3D LGE-CMR short-axial slice.** From the 3D LGE-CMR (left), the short axis was segmented using a semi-automated methodology, as described in previous publications.^3,8^ From the resultant myocardial volume, scar and infarct border zone were identified using the full-width half max method (right), as described in previous works.^3,9^


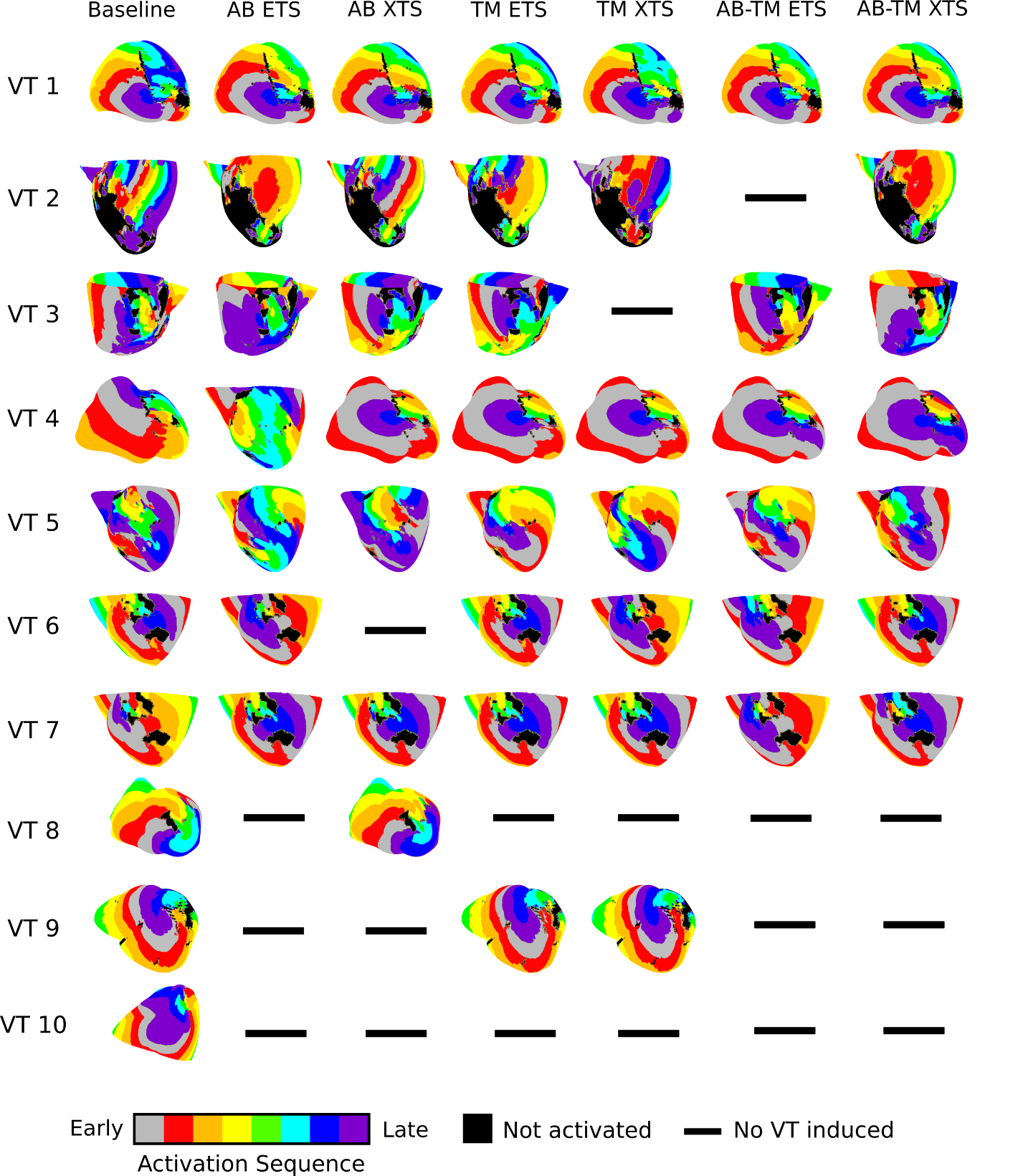


**Supplemental Figure 2: Summary of all induced VTs across all models and stimulus protocols.** Each row corresponds to a given VT and each column corresponds to a given model and corresponding stimulus protocol. If a dashed line is shown, this means that the stimulus protocol did not induce VT in that given model. VT: ventricular tachycardia, ETS: earliest timed stimulus, XTS: exact timed stimulus, AB: apicobasal, TM: transmural, AB-TM: combined apicobasal and transmural

**References**

1. ten Tusscher, K. H. W. J. & Panfilov, A. v. Alternans and spiral breakup in a human ventricular tissue model. *American Journal of Physiology - Heart and Circulatory Physiology* **291**, (2006).

2. Prakosa, A. *et al.* Personalized virtual-heart technology for guiding the ablation of infarct-related ventricular tachycardia. *Nature Biomedical Engineering* **2**, 732–740 (2018).

3. Arevalo, H. J. *et al.* Arrhythmia risk stratification of patients after myocardial infarction using personalized heart models. *Nature Communications* **7**, (2016).

4. Sung, E. *et al.* Personalized Digital-Heart Technology for Ventricular Tachycardia Ablation Targeting in Hearts with Infiltrating Adiposity. *Circulation: Arrhythmia and Electrophysiology* (2020) doi:10.1161/CIRCEP.120.008912.

5. Deng, D., Prakosa, A., Shade, J., Nikolov, P. & Trayanova, N. A. Sensitivity of Ablation Targets Prediction to Electrophysiological Parameter Variability in Image-Based Computational Models of Ventricular Tachycardia in Post-infarction Patients. *Frontiers in Physiology* **10**, 628 (2019).

6. Deng, D., Prakosa, A., Shade, J., Nikolov, P. & Trayanova, N. A. Characterizing Conduction Channels in Postinfarction Patients Using a Personalized Virtual Heart. *Biophysical Journal* (2019) doi:10.1016/j.bpj.2019.07.024.

7. Vigmond, E. J., Aguel, F. & Trayanova, N. A. Computational techniques for solving the bidomain equations in three dimensions. *IEEE Transactions on Biomedical Engineering* **49**, 1260–1269 (2002).

8. Prakosa, A. *et al.* Methodology for image-based reconstruction of ventricular geometry for patient-specific modeling of cardiac electrophysiology. *Progress in Biophysics and Molecular Biology* **115**, 226–234 (2014).

9. Schmidt, A. *et al.* Infarct Tissue Heterogeneity by Magnetic Resonance Imaging Identifies Enhanced Cardiac Arrhythmia Susceptibility in Patients With Left Ventricular Dysfunction. *Circulation* **115**, 2006–2014 (2007).
